# Ileocecal knotting as a rare cause of small bowel obstruction: a case report

**DOI:** 10.1097/MS9.0000000000001800

**Published:** 2024-02-28

**Authors:** Nishanta Dallakoti, Ved Prakash Pant, Nabin Pokhrel, Prashant Bhatta, Vijay Kumar Rana, Rupesh Prasad Sah

**Affiliations:** a B.P. Koirala Institute of Health Sciences; b Department of Surgery, B.P. Koirala Institute of Health Sciences, Dharan, Nepal

**Keywords:** bowel obstruction, case report, ileocecal knotting, intestinal knot syndrome

## Abstract

**Introduction:**

Ileocecal knot syndrome, a rare cause of small bowel obstruction where the ileum wraps around the cecum, poses a significant challenge for preoperative diagnosis. Prompt intervention is crucial due to the risk of rapid bowel deterioration and increased mortality.

**Case presentation:**

A 45-year-old female presented with central abdominal pain associated with vomiting, abdominal distension, and obstipation. On examination, she was ill-looking with hypotension, tachycardia with a feeble pulse, direct and rebound abdominal tenderness, and absent bowel sounds. Aggressive fluid resuscitation was done. Based on the clinical presentation and abdominal radiograph suggestive of intestinal obstruction, an emergency exploratory laparotomy was done, which showed an ileocecal knot and 130 cm of gangrenous ileum. Peritoneal lavage followed by resection of non-viable ileum with double barrel ileostomy was done.

**Discussion:**

Ileosigmoid, appendico-ileal, ileoileal, and ileocecal knotting are the various types of intestinal knotting, with very few cases of ileocecal knotting being reported. Intestinal knotting causes severe bowel obstruction, resulting in reduced mucosal perfusion, progressive ischemia, and peritonitis, leading to high mortality. X-ray findings of multiple air-fluid levels are non-specific, and for definitive diagnosis, laparotomy is required. Assessing bowel viability before definitive surgery is essential. Despite positive outcomes, extensive resection can lead to malabsorption and ileus, with potential risk for developing short bowel syndrome.

**Conclusion:**

Despite its rarity, the possibility of ileocecal knotting should be considered in cases of small bowel obstruction due to its potential for rapid deterioration. Prompt resuscitation followed by emergency laparotomy is necessary to prevent mortality.

## Introduction

Highlights

- Ileocecal knotting is a rare cause of small bowel obstruction.
- Rapid deterioration of the patient due to progressive bowel ischemia and necrosis.
- Precise preoperative diagnosis of ileocecal knotting is challenging.
- Early intervention with prompt and aggressive management is necessary.

Bowel obstruction is a common cause of acute abdominal presentations, with small bowel obstruction constituting the majority of cases^1,2^. Frequently encountered causes of small bowel obstruction include adhesions, hernias, neoplasms, Crohn’s disease, and small bowel volvulus^3^. Twisting of a loop of bowel with an intervening knot, described as intestinal knot syndrome, is a rare cause, with the even rarer subset being the ileocecal knot, where a loop of ileum wraps over the cecum and ascending colon^4–6^. This case has been reported in line with SCARE (Surgical CAse REport) 2023 guidelines^7^.

While certain clinical and radiological findings may offer clues, precise preoperative diagnosis of ileocecal knotting remains challenging. There is a rapid deterioration of the patient due to progressive bowel ischemia and necrosis, resulting in increased morbidity and mortality^3,6^. Thus, early intervention with prompt and aggressive management is necessary. Here, we present a case of ileocecal knotting presenting with small bowel obstruction.

## Case presentation

A 45-year-old female presented to the emergency department of a tertiary care hospital with severe, non-radiating central abdominal pain for one day, associated with non-bilious vomiting related to meals, abdominal distension, and inability to pass stool and flatus. A lower-segment cesarean section was performed 15 years back. There is no history of abdominal trauma, fever, cough, chest pain, or prior medical illness. On examination, she was conscious and ill-looking with a feeble pulse of 114 beats per minute and blood pressure of 80/50 mmHg. The abdomen was moderately distended with direct and rebound tenderness and absent bowel sounds.

**Figure 1:**
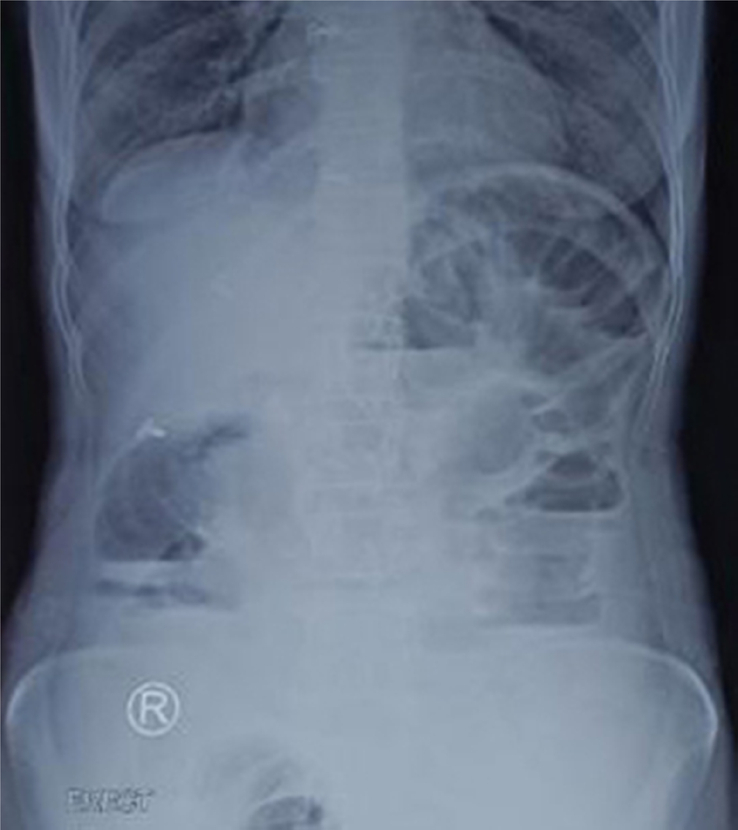
Plain abdominal X-ray showing multiple air-fluid levels and dilated bowel loop suggestive of small bowel obstruction.

**Figure 2:**
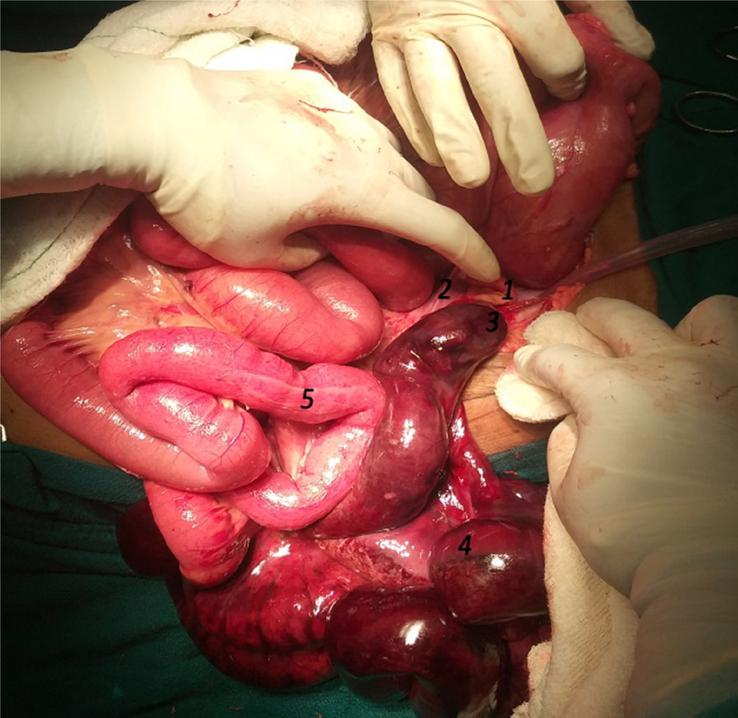
Intraoperative photograph: 1. Appendix, 2. Band, 3. Ileocecal junction, 4. Gangrenous ileum, 5. Proximal ileum.

Aggressive fluid resuscitation was done with 3 l of normal saline via two wide-bore cannulas, and her blood pressure improved to 110/70 mmHg. Nasogastric decompression and transurethral catheterization were then performed. Abdominal radiograph revealed multiple air-fluid levels and dilated bowel loops, suggestive of acute intestinal obstruction (Fig. 1). Emergency exploratory laparotomy was performed, which showed a loop of ileum wrapping around the cecum forming a knot, and a segment of gangrenous ileum, measuring 130 cm in length, extending proximally from 5 cm proximal to the ileocecal junction (Fig. 2). Hemorrhagic ascitic fluid was noted. Peritoneal lavage with untwisting of the knot and warm mopping of the gangrenous ileum were done. The non-viable segment was then resected, and a double barrel ileostomy was created. The abdomen was closed with a drain in situ.

Postoperatively, the patient was transferred to the intensive care unit (ICU) and upon improvement, transferred to the ward on the fourth postoperative day, where she was managed with intravenous fluids, analgesics, and antibiotics. The drain was removed on the 10th postoperative day. The patient was discharged on the 12th postoperative day with a stoma bag. At the time of discharge, the patient was hemodynamically stable with no abdominal pain or distension, was tolerating oral intake, and the stoma was functioning. Skin stapler removal was performed during a follow-up visit 10 days after hospital discharge.

## Discussion

Intestinal knot syndromes are rare conditions in which bowel loops twist over one another, leading to the rapid deterioration of symptoms and a progressive loss of bowel viability^5,6^. Various types of knotting, such as ileosigmoid, appendico-ileal, ileoileal, and ileocecal knotting, have been reported, with ileosigmoid knotting being the most common^4–6^. In our patient, the operative findings were suggestive of ileocecal knotting, which is even a rarer variant. To the best of our knowledge, only three cases have been reported previously^4,6,8^ (Table 1).

**Table 1:** Literature review and case study summary of demographic, clinical presentation, X-ray findings, and treatment.

Serial number	Case	Age/sex	Presentation	X-ray abdomen	Treatment
1.	Roy *et al*.,^8^	15/M	Subacute intestinal obstruction followed by acute intestinal obstruction	Multiple air-fluid levels	Resection of devitalized ileum and end ileostomy
2.	Gebresellassie,^6^	21/M	Hypovolemic shock, abdominal cramps, bilious vomiting, abdominal distension, absent bowel sounds	Multiple air-fluid levels and distended bowel loops	Aggressive resuscitation, exploratory laparotomy, resection of the gangrenous segment, and anastomosis of jejunum to transverse colon
3.	Menon *et al*.,^4^	72/F	Vomiting, right iliac fossa pain, grossly distended abdomen, and absent bowel sounds	Fecal loading, dilated cecum, and ascending colon	Emergency laparotomy followed by right hemicolectomy and small bowel resection
4.	Our case	45/F	Hypovolemic shock, acute abdominal pain, vomiting, abdominal distention, obstipation, and absent bowel sounds	Multiple air-fluid levels and dilated bowel loops	Aggressive resuscitation and exploratory laparotomy with peritoneal lavage, warm saline moping, resection of gangrenous ileum, and double barrel ileostomy

Intestinal knots are more frequently observed in African and Asian populations and are comparatively rarer in the West. Potential contributing factors include a high-fiber diet following prolonged fasting, a diet rich in bulk, a mobile cecum, and a cecum attached to a long mesentery^2,4,9,10^. In our case, the patient’s consumption of a fiber-rich vegetarian diet might be a possible contributing factor.

An ileocecal knot is formed through the combination of cecal volvulus and ileosigmoid knotting. This condition involves the cecum twisting along the mesentery, followed by the wrapping of the ileum around the twisted cecum^4^. Intestinal knotting results in an advanced bowel obstruction characterized by an increase in intramural pressure and hypovolemia. This leads to decreased mucosal perfusion, progressive bowel ischemia, and necrosis, subsequently causing peritonitis within a short time and resulting in high mortality^3,6^.

Intestinal knot syndrome presents with symptoms of bowel obstruction, including abdominal pain, nausea, vomiting, distention, constipation, and absent bowel sounds, and may even lead to shock. It is often challenging to diagnose preoperatively^3,4,6^. An X-ray may reveal multiple air-fluid levels but lacks specificity for the diagnosis. A computed tomographic scan is the best investigation modality, which shows a swirling of small bowel mesentery adjacent to a transition zone in the adjacent colon. A definitive diagnosis can be made only after laparotomy^4^. In our case, due to the patient’s late presentation, we performed an emergency laparotomy based on clinical and X-ray findings. It revealed an ileocecal knot and a gangrenous ileum.

Evaluating bowel segment viability is essential before a definitive surgical procedure. Bowel resection and anastomosis typically yield superior results, with postoperative care being of utmost importance^2,10^. In our case, following viability assessment, we resected 130 cm of the ileum and created a double barrel ileostomy with a plan for a secondary anastomosis. Due to extensive bowel resection and the associated risk of malabsorption and functional ileus, the patient received postoperative care in the ICU. Patients with resection of a large segment of the bowel are prone to develop short bowel syndrome^6^. In our case, the patient’s condition improved, and there were no complications during the 2-month postoperative follow-up.

## Conclusion

Ileocecal knotting, although rare, should be considered in cases of small bowel obstruction due to its potential for rapid progression to bowel ischemia, necrosis, and subsequent peritonitis, which can result in patient mortality. Vigorous resuscitation, followed by emergency laparotomy, is essential for accurate diagnosis and preventing further deterioration of the patient, ultimately reducing mortality risk.

## Ethical approval

Ethical approval is not required for this case report.

## Consent

Clear and written informed consent was obtained from the patient for the publication of this case report and accompanying images. A copy of written consent is available for review by the editor-in-chief of this journal on request.

## Sources of funding

This research did not receive any specific grant from funding agencies in public, non-commercial, or non-profit sectors.

## Author contribution

N.D., V.P.P., and N.P.: conceptualization, resources, visualization, writing original draft, writing review and editing, and final approval of the manuscript; P.B.: conceptualization, resources, writing review and editing, and final approval of the manuscript; V.K.R. and R.P.S.: conceptualization, resources, writing review and editing, and final approval of the manuscript.

## Conflicts of interest disclosure

There are no conflicts to be declared.

## Research registration unique identifying number (UIN)

1. Name of the registry: not applicable.
2. Unique identifying number of the study: not applicable.
3. Hyperlink to your specific registration (must be publicly accessible and will be checked): not applicable.

## Guarantor

Nishanta Dallakoti.

## Data availability statement

Not applicable.

## Provenance and peer review

Not commissioned, externally peer-reviewed.
